# Multiple Cystic Brain Infection: A Diagnostic Dilemma of Neurocysticercosis and Intracranial Tuberculoma

**DOI:** 10.7759/cureus.66231

**Published:** 2024-08-05

**Authors:** Jia Xin Chew, Juen Kiem Tan, Xiong Khee Cheong, Wen Chung Ho, Noorasyikin Mohamed Arifin, Suganthi Chinnasami, Md Hanif Md Arif, Lim Kah Chuan, Shaharudeen Kamaludeen

**Affiliations:** 1 Department of Internal Medicine, Hospital Sultan Idris Shah, Serdang, Selangor, MYS; 2 Faculty of Medicine, Universiti Kebangsaan Malaysia Medical Centre, Kuala Lumpur, MYS; 3 Department of Medicine, Hospital Canselor Tuanku Muhriz, Kuala Lumpur, MYS; 4 Department of Medicine, Universiti Putra Malaysia, Selangor, MYS; 5 Department of Medicine, Hospital Kuala Lumpur, Kuala Lumpur, MYS; 6 Department of Radiology, Hospital Kuala Lumpur, Kuala Lumpur, MYS

**Keywords:** scolex, taenia solium, ncc, tuberculoma, neurocysticercosis

## Abstract

Neurocysticercosis (NCC) is a central nervous system infection caused by *Taenia solium* or pork tapeworm. It affects millions worldwide and represents a leading cause of epilepsy in developing countries. NCC may be challenging to distinguish from intracranial tuberculomas, with tuberculosis being highly prevalent in developing countries. We highlight the importance of clinical history, including exposure history and neuroimaging, in obtaining an accurate diagnosis to enable prompt treatment. This report presents the case of a 26-year-old man diagnosed with NCC and presenting with acute giddiness and headache. Otherwise, there was no history of fever or constitutional symptoms. Neuroimaging demonstrated multiple cerebral lesions over both hemispheres, with degenerating scolex on brain MRI. He recovered well following a combination of oral albendazole, praziquantel, and corticosteroids. This case highlights the salient features that distinguish NCC from intracranial tuberculoma. Early and precise diagnosis will ensure that patients receive optimal treatment, expedite recovery, and prevent further complications.

## Introduction

Neurocysticercosis (NCC) refers to an infection of the central nervous system caused by cysticerci or larvae of the parasite *Taenia solium* [1]. Cysticercosis has been classified as a neglected tropical illness by the World Health Organization (WHO) and affects an estimated 2.56 to 8.30 million individuals. Additionally, research indicates that approximately one-third of people with epilepsy residing in endemic regions are diagnosed with NCC [2]. Bizhani et al.'s systematic review and meta-analysis found that its prevalence in Southeast Asia ranges from 0.80% in Indonesia to 41.8% in Thailand, and globalization has boosted prevalence in formerly low-prevalence areas [3]. The overall incidence of NCC in Malaysia appears low due to underdiagnosis [4]. This case study raises awareness concerning NCC, enabling patients' access to prompt and suitable therapy to mitigate its adverse consequences.

This article was previously presented as a meeting abstract at the 30th Annual Scientific Meeting of the Malaysian Society of Neurosciences on October 1, 2022. It was also published on the Research Square preprint server on December 11, 2023.

## Case presentation

A 26-year-old man presented with giddiness for four days. It was acute in onset, first noticed while he was driving. It was associated with a continuous mild generalized headache with no postural or diurnal variation. He did not experience any fever, change in appetite, weight loss, or night sweats. He had a history of miliary tuberculosis and pneumothorax in 2017, which was treated with antituberculous drugs. He did not have a family history of malignancy. He was clinically oriented on examination, pupils were equal and reactive, he was afebrile, and vital signs were within normal limits. A complete neurological examination indicated no motor or sensory deficit, cerebellar signs, or cranial nerve defects. Subsequent systemic examination, including genital examination, did not reveal any abnormalities.

**Figure 1:**
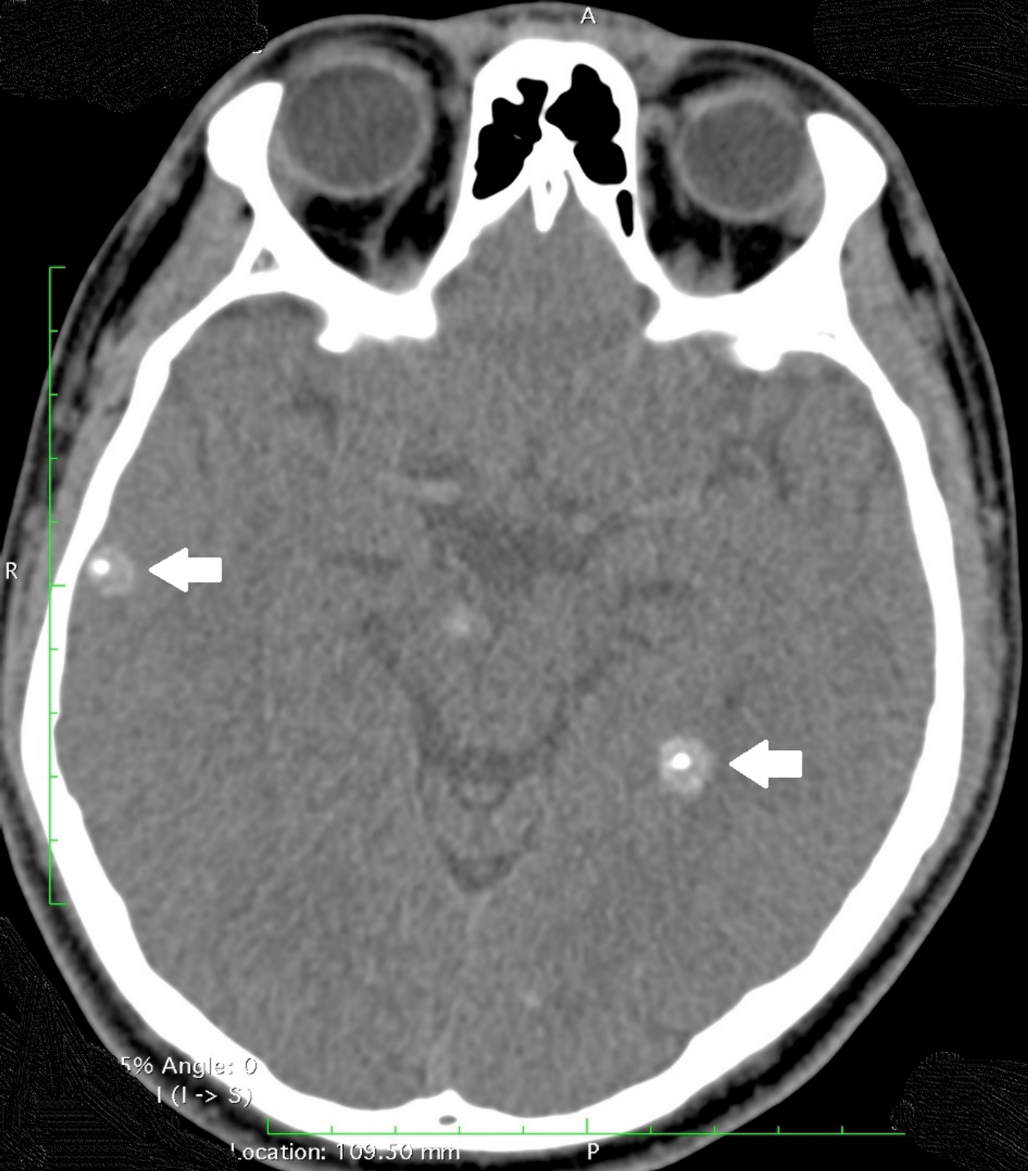
Plain CT of the brain, axial cut, showing calcified scolex within the cyst (thick arrow), giving a “target appearance.”

**Figure 2:**
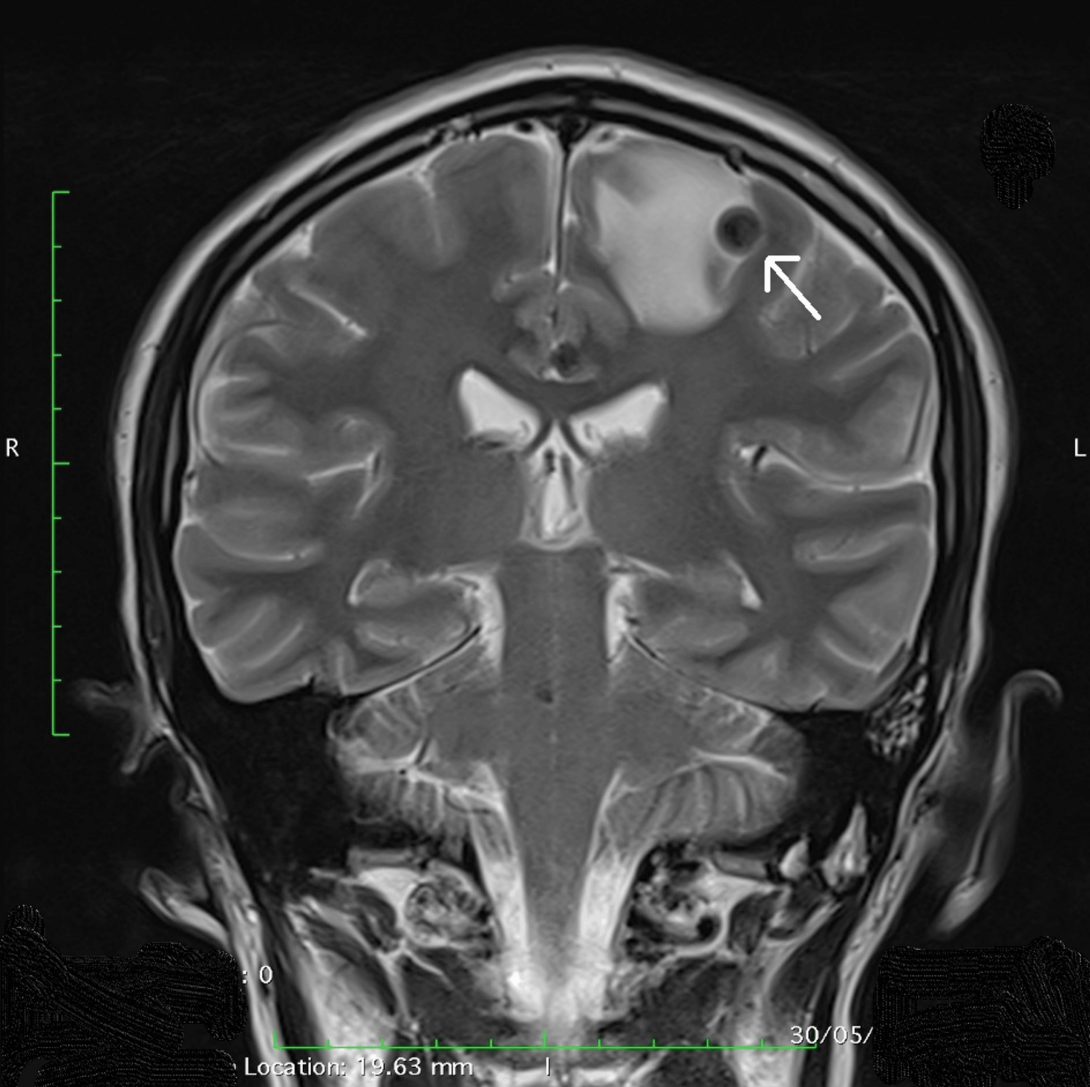
MRI of the brain, T2-weighted coronal section, showing left frontal cyst with hypointense cystic wall and significant perilesional edema (thin arrow).

**Figure 3:**
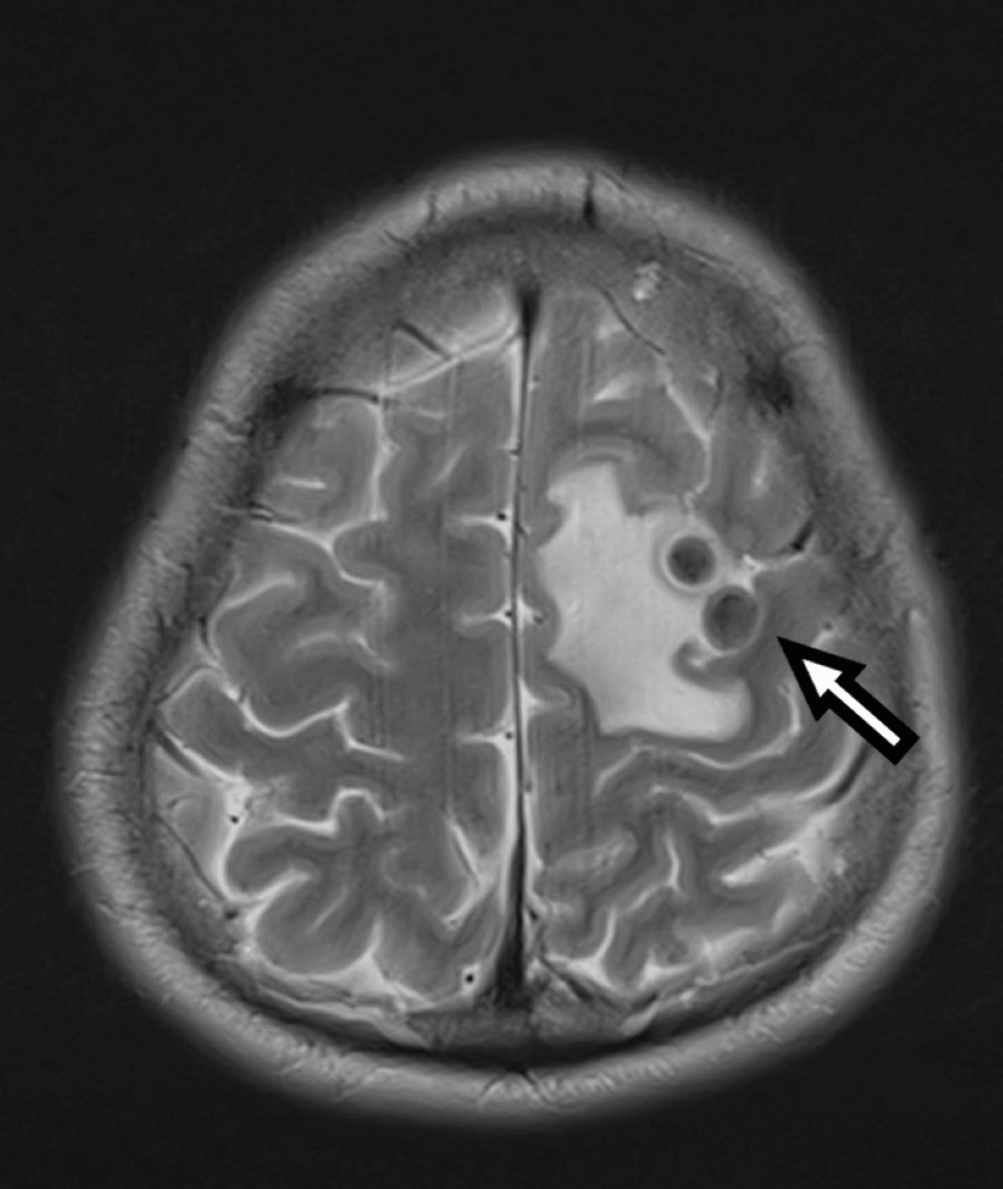
MRI of the brain, T2-weighted axial section, showing left frontal cysts with hypointense cystic wall with significant surrounding perilesional edema (white arrow). The left frontal cysts are similar to those in Figure 2.

**Figure 4:**
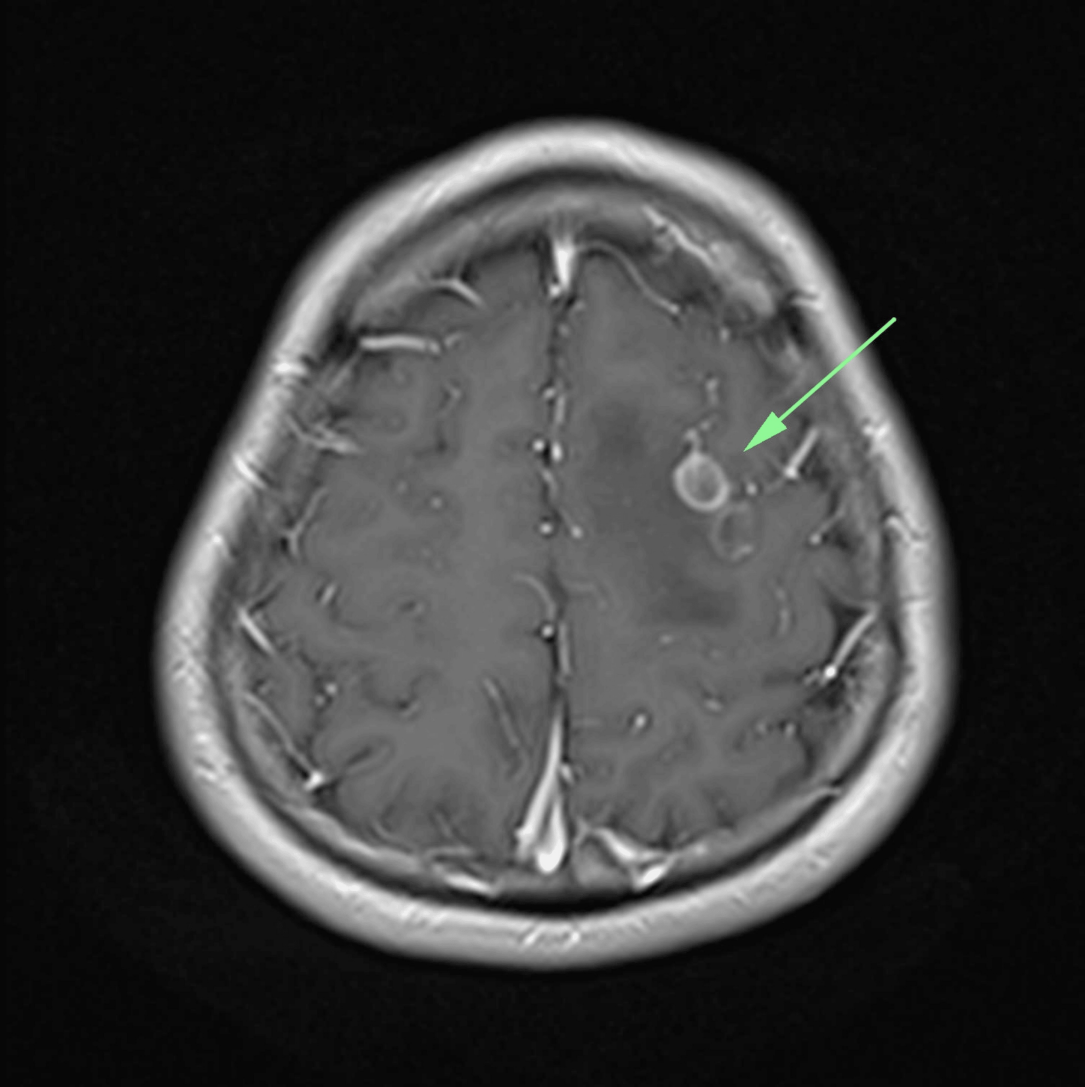
MRI of the brain, T1-weighted post-contrast axial section, showing left frontal cysts with varying degrees of peripheral enhancement and thickness of cystic wall (green arrow).

**Figure 5:**
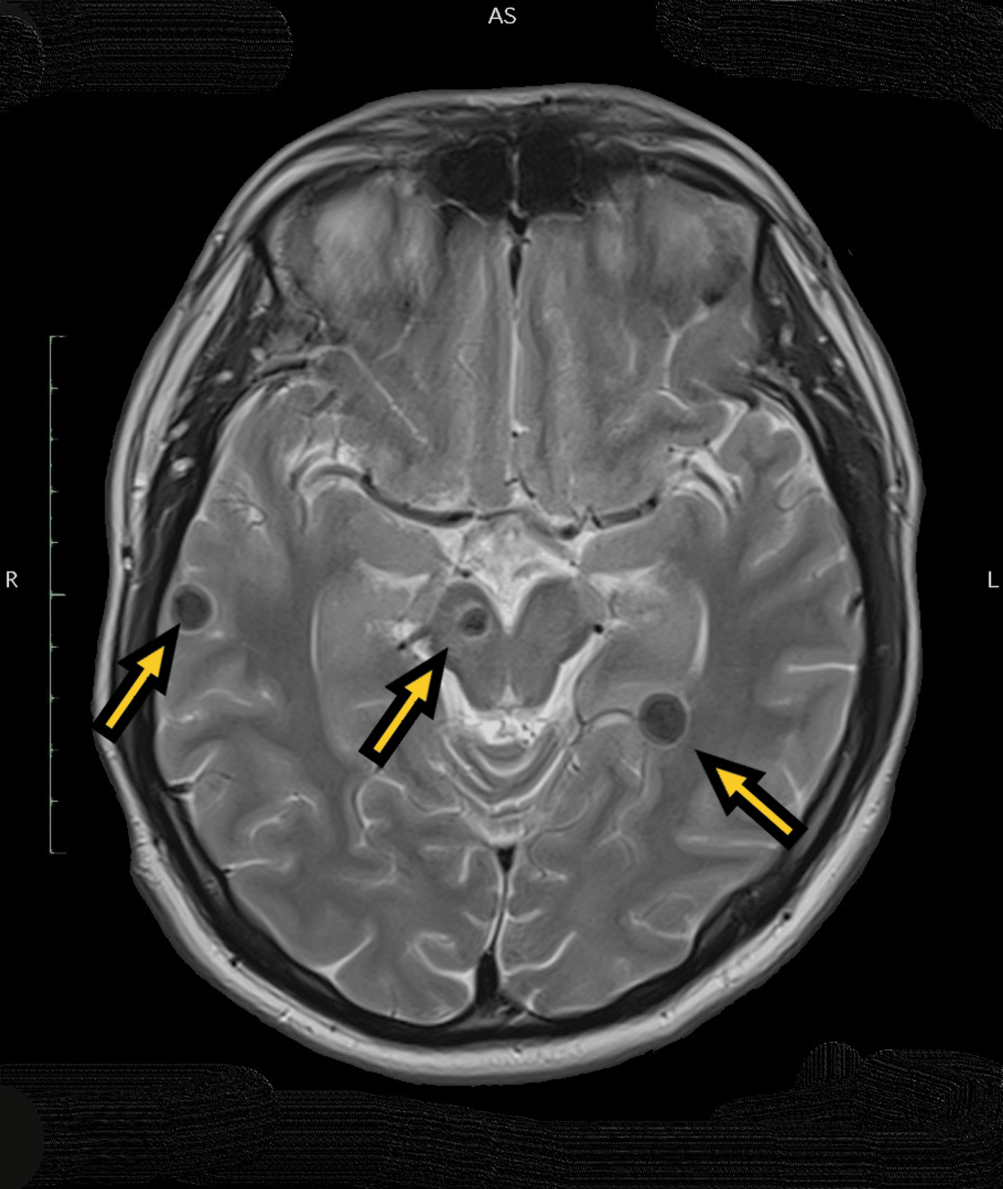
MRI of the brain, T2-weighted axial section, showing bilateral temporal and right midbrain cysts with hypointense cystic wall and minimal perilesional edema (yellow arrow).

Complete blood count, and renal and liver profile were unremarkable. Inflammatory and tumor markers were not elevated. Chest radiograph demonstrated reticulonodular opacities over both lung fields. Workup for tuberculosis, including sputum for acid-fast bacilli, culture and sensitivity, and PCR, were negative. Computed tomography (CT) of the brain demonstrated scattered hyperdense nodules with minimal perilesional edema over both temporal lobes and two nodules at the left vertex. Lumbar puncture revealed a slightly elevated opening pressure of 30 cmH_2_O, and cerebrospinal fluid (CSF) protein was 312 mg/L and acellular. Viral screening, toxoplasma IgM/IgG, and rapid plasma reagin were negative. Magnetic resonance imaging (MRI) of the brain demonstrated multiple bilateral cerebral and brainstem rim-enhancing lesions, the presence of scolex within the cyst, and perilesional edema over the left vertex. Similar cystic lesions were noted over both hemispheres with varying degrees of edema, few with calcified components within, signifying different stages of the disease. The CT and MRI images are shown in Figures 1-5.

Further history suggested consumption of undercooked pork. A diagnosis of NCC, in varying stages of disease (colloidal vesicular and vesicular nodular stage), was made, and he was initiated on oral albendazole and praziquantel for two weeks. In addition, he was prescribed T. dexamethasone 7mg once daily (0.1mg/kg/day) for two weeks, which was then tapered off gradually over a week. He was well after completing treatment with no complications.

## Discussion

The life cycle of *Taenia solium*, or pork tapeworm, is complex. Humans play a role as the only definitive host, while pigs represent intermediate hosts. The infection starts with the ingestion of vegetation contaminated with eggs or gravid proglottids by intermediate hosts. The oncospheres hatch and penetrate the small intestine, circulating in the bloodstream before they settle and grow in the host’s striated muscles to form cysticerci. Furthermore, consuming contaminated undercooked pork results in the ingestion of cysticerci that eventually mature into adult tapeworms while remaining attached to the small intestine. Infection of human hosts with any species of taenia (*T. solium*, *T. saginata*) is termed “taeniasis” [2].

Matured tapeworms produce proglottids and eggs that pass out in feces to complete the cycle. NCC differs from taeniasis as it is transmitted via the fecal-oral route when the definitive host ingests food containing *T. solium* eggs. The oncosphere invades the host’s intestinal wall once the egg’s coating disintegrates when exposed to digestive enzymes. In the bloodstream, the oncospheres migrate to various organs, including the brain [2]. Our patient may have contracted NCC through autoinfection or food contaminated by other infected hosts. Unlike *T. solium*, *T. saginata* infection does not lead to NCC. It usually causes gastrointestinal symptoms, as its length is two to three times more than *T. solium*. Migration of proglottids in these species may lead to cholangitis or appendicitis [5].

NCC typically presents as seizures or signs of elevated intracranial pressure such as headache or giddiness. Clinical presentation is dictated by the volume of cysticerci, stage of infection, and location of the affected area. While the symptoms mentioned earlier are associated with parenchymal NCC, ventricular NCC or subarachnoid NCC can lead to obstructive hydrocephalus or meningitis, respectively [6]. Seizures, being the most prevalent symptom, are likely related to inflammatory reactions and processes. The degeneration of cysts triggers an inflammatory response that acts on intracellular parasites. This leads to a surge of pro-inflammatory cytokines that may disrupt the blood-brain barrier and glymphatic system, leading to significant perilesional edema [7]. Eventually, calcified cysts formed as a sequela to the infection may cause enduring predilection for seizures and developing epilepsy.

NCC can be classified based on the location of the lesion as parenchymal NCC (brain parenchyma) or extraparenchymal NCC such as ventricular, subarachnoid, meningeal, or spinal NCC [8]. These lesions may have mixed presentations and are not necessarily confined to a particular location. NCC can also be represented in four stages: vesicular, colloidal vesicular, granular nodular, and nodular calcified stage. The vesicular stage is characterized by viable parasites appearing as marginal nodules and projecting into a cyst. As they express various molecules that aid in avoiding detection from the host’s immune system, there is little to no surrounding inflammatory reaction. CT imaging will demonstrate the thin-walled cysts with eccentrically located scolex. The scolex may appear iso- or hyperintense on T1- or T2-weighted images and could be visualized better through the fluid-attenuated inversion recovery (FLAIR) and diffusion-weighted images (DWI) sequences [9].

Cystic lesions in the vesicular stage may persist for months to years before they move on to the colloidal vesicular stage. The larvae undergo degeneration, and the cyst reduces in size. Surrounding inflammation leads to irregularity and thickening of the cyst wall, which enhances with contrast on CT imaging. In addition, the cyst's contents may appear hyperdense at this stage. MRI of the brain is useful in supporting the diagnosis of NCC at this stage, with marked hyperintensity in T2-weighted and FLAIR images with significant perilesional edema. The scolex may be absent in later stages as the cyst continues to degenerate. MR spectroscopy might differentiate the lesions from others such as malignancies, as NCC demonstrates elevated choline, lactate, lipid, and decreased N-acetylaspartate and creatine [9]. As the edema peaks at this point, most patients develop symptoms and present during this period.

The third stage is the granular nodular stage, whereby the degeneration of the cyst progresses to the point where it appears nodular, small with a ring-like enhancement, albeit less hyperintense in imaging than previous stages. The surrounding perilesional edema resolves and is replaced with pericystic gliosis. The fourth and final stage is the nodular calcified stage, where the cysts are non-active and completely mineralized, with no surrounding edema. It is best visualized on CT imaging with the appearance of a calcified nodule. Despite its non-active form, it predisposes patients to seizures, possibly due to the antigenic properties of its remnants or due to its resulting structural lesions [7,9].

A detailed history-taking and physical examination is important to identify risks, such as travel history, poor sanitation, access to clean water, contact with potential hosts, suboptimal hygiene practices, and location (endemicity) [10]. Subsequently, imaging modalities such as CT and MRI brain are essential to assist in diagnosis and ascertain the stage of disease, especially in centers without access to serological tests such as enzyme-linked immunoelectrotransfer blot (EITB) test for *T. solium*. Our patient’s initial CT of the brain displayed hyperdense foci with minimal perilesional edema surrounding the cystic contents over the temporal regions. The MRI of the brain demonstrated significant pericystic edema over the left frontal lobe, as well as thickened cystic wall with focal nodular thickening, likely representing a degenerating scolex. Therefore, radiologically, our patient’s disease shows a mixed pattern of late colloidal vesicular and granular nodular stage.

Intracranial tuberculoma may resemble NCC radiologically. However, there are several differentiating features. While NCC usually appears cystic with a scolex, caseating tuberculomas comprise three layers. In MRI of the brain, the inner caseous necrosis layer is iso- or hypointense on T1- and T2-weighted images, with FLAIR imaging showing extensive necrosis. The middle layer shows heterogenous hypo- and hyperintense signals with gadolinium enhancement on T1- and T2-weighted imaging. Finally, the external layer represents the collagenous capsule, appearing iso- or hypointense [11].

On the other hand, non-caseating tuberculomas display mild hypointensity on T1-weighted images, while hyperintensity is observed on T2-weighted images. In addition, tuberculomas are more likely to cause meningeal enhancement than parenchymal NCC. MR spectroscopy also assists in discerning between both tuberculomas and NCC. Intracranial tuberculoma demonstrates marked lipid and lactate peaks in up to 86% of cases, while NCC tends to have accompanying choline peaks along with various amino acids [11,12].

Our patient was treated with oral albendazole and praziquantel for two weeks. This is in line with recommendations by the Infectious Diseases Society of America (IDSA), where combination therapy was superior to albendazole monotherapy in radiological resolution of lesions [10]. Fundoscopic eye examination is essential to identify intraocular cysticerci prior to treatment, as antiparasitic agents may cause significant local inflammation due to the degradation of cysts, resulting in visual impairment or blindness. Prescription of anti-inflammatory agents or corticosteroids concomitantly reduces such risks and hastens recovery. In the setting of extraparenchymal NCC causing hydrocephalus, the elevated intracranial pressure should be resolved prior to the initiation of antiparasitic agents. Clinical improvement should be monitored with repeat imaging semi-annually until complete resolution is observed [10].

## Conclusions

Distinguishing between NCC and intracranial tuberculoma can be challenging. This case demonstrates the importance of thorough history-taking and neuroimaging modalities, particularly MRI of the brain, to support the diagnosis of NCC, whose features may overlap with those of tuberculomas. The clinical presentation and the varying stages of disease observed in the MRI brain point towards the diagnosis of NCC instead of other lesions with similar presentations. Identifying the stage of the disease based on its imaging features assists in determining the activity of the disease as well. Prompt identification and treatment ensure a good clinical outcome, as management would differ greatly between the conditions.
